# The relationship between educational attainment, lifestyle, self-rated health, and depressive symptoms among Chinese adults: a longitudinal survey from 2012 to 2020

**DOI:** 10.3389/fpubh.2024.1480050

**Published:** 2024-12-04

**Authors:** Xinyan Xiong, Rita Xiaochen Hu, Wenyuan Ning

**Affiliations:** ^1^School of Sociology, Huazhong University of Science and Technology, Wuhan, China; ^2^Crown Family School of Social Work, Policy, and Practice, University of Chicago, Chicago, IL, United States; ^3^School of Marxism, Zhongnan University of Economics and Law, Wuhan, China

**Keywords:** educational attainment, sleep time, exercise time, overtime, leisure time, housework time, self-rated health, depressive symptoms

## Abstract

Education holds significant implications for individual health. This work aims to examine the relationship between educational attainment, lifestyle, self-rated health, and depressive symptoms among Chinese adults. We used China Family Panel Studies data from 2012 to 2020. Multiple linear regression models were used to explore the relationship between educational attainment and self-rated health and depressive symptoms, where multidimensional lifestyle (sleep time, exercise time, overtime time, leisure time, housework time, and protein intake) was the mediator variable of the above relationship, and the causal step method was used to test the mediating effect. Our findings show that educational attainment is associated with higher levels of self-rated health and lower levels of depressive symptoms. More importantly, educational attainment also indirectly affects individuals’ self-rated health and depressive symptoms through lifestyle. These findings reveal health interventions to develop education further and improve its quality.

## 1 Introduction

Health is an essential concern for the world, as it constitutes a vital foundation for ensuring individuals’ quality of life and overall well-being (1, 2). Education plays a critical role in shaping health inequalities because it is a fundamental determinant of an individual’s occupation and income, which can have long-lasting impacts on health (3, 4).

However, research findings on the relationship between education and health have not yielded a consensus. Some studies indicate a positive correlation, thus establishing an educational gradient in health (5, 6). Conversely, others have discovered negative or non-significant associations (7, 8). Furthermore, although previous studies have examined the importance of health behaviors, such as smoking and alcohol consumption, in the education-health relationship (9, 10), the research on lifestyle as a mediating mechanism is less comprehensive. Most importantly, much research has been conducted in Western countries, with limited studies across social, political, and economic contexts, thus necessitating further in-depth exploration.

### 1.1 Educational attainment and health

Education plays a crucial role in health stratification (11). Researchers have shown the positive impact of education on physical and mental health (11). Individuals with higher levels of education exhibit significant health advantages compared to those with lower levels of education (12), such as lower mortality rates (13), reduced incidence of hypertension (14), higher self-rated health (15), and lower risk of depression (16).

Despite the widely established notion that higher education levels are a stable predictor of favorable health outcomes (12), some scholars have expressed skepticism regarding the positive relationship between educational attainment and health. Some studies show that individuals with higher education did not have a statistically significant advantage in self-rated health and reduced depression (17, 18). For instance, Bracke et al.’s research revealed that individuals with higher levels of education were more likely to experience mental health issues such as depression (7, 19).

While a strong correlation between educational attainment and health has been extensively established (6, 7, 11, 19), some scholars argue that the role of educational attainment in influencing individual health is limited. For example, Veenstra (20) found no significant causal relationship between educational attainment and health status among individuals under 39. The current study aims to harness longitudinal data across 10 years on Chinese adults to examine the associations between educational attainment and health.

### 1.2 Lifestyle as a mediator

#### 1.2.1 Educational attainment and lifestyle

Similar to the educational gradient in health, there is also an educational gradient in health behaviors; well-educated individuals tend to develop healthier lifestyles (21, 22). There is also socioeconomic inequality in dietary habits (23). Individuals with higher levels of education tend to consume relatively more lean meat and low-fat dairy products (24). Another study suggested that people with higher educational attainment are more likely to follow the Nordic Nutrition Recommendations (NNR 2012), meaning they are more likely to consume protein as recommended (23). A systematic review shows that individuals with higher cultural capital are less likely to smoke and have a higher propensity to eat healthier and engage in physical activity (25).

However, as lifestyle is closely related to specific cultural norms (26), the strong positive correlations described above may show opposite trends in different cultural contexts. In contrast to Western societies, a comparative study of Chinese and American samples found that unhealthy behaviors (e.g., imbalanced diet, low physical activity, smoking, and alcohol consumption) are more prevalent among individuals with higher levels of education and income in China (27). Regarding working hours, a recent study indicates that upper-class women are less likely to spend time working during non-standard hours (e.g., weekends) (28). Another study found that people with lower levels of education spent more time watching television due to fewer opportunities to choose leisure activities (29). However, Farrell’s study suggested that there might not be a sustained causal link between educational attainment and health behaviors, such as smoking (30). The relationship between educational attainment and health behaviors has yet to reach a consensus.

#### 1.2.2 Lifestyle and physical and mental health

A healthy lifestyle is a crucial pathway to achieving health, and extensive research has confirmed a positive correlation between health behaviors and health status (22, 31, 32). Empirical studies have shown that alcohol consumption (9), lack of exercise (9), sleep deprivation (9), prolonged overtime work (33), and extended housework (34) all have significant adverse effects on health. Among them, overtime work was considered an important risk factor for mental disorders. A comparative study found that anxiety and depression rates were significantly higher among overtime workers (35) compared to workers with normal working hours. Furthermore, inadequate sleep (<7 h) may be a risk factor for poorer self-rated health and depressive symptoms in Chinese older individuals (34, 36). Several studies supported a close relationship between leisure activity participation and health (37, 38). Research showed that participation in sedentary leisure activities, such as watching television and engaging in crafts, played a significant role in reducing depressive emotions and enhancing psychological well-being, but increased the risk of developing type 2 diabetes and cardiovascular disease (37). The relationship between housework time and health is complex, with some studies suggesting that prolonged housework implies a “burden” in an individual’s life, which may lead to a higher risk of physical symptoms, illness, self-rated poor health, and mental disorders (34, 39–41). Some researchers also suggest that housework is a form of physical activity, similar to sports and exercise, that has a positive role in maintaining health and is associated with reduced mortality and adverse mental health (42). In addition, protein intake is considered to contribute to the improvement of physical function (43), thereby promoting overall health and well-being. Empirical studies on the older adults found that higher levels of protein intake were associated with a greater likelihood of healthy aging, including a lower probability of chronic diseases and physical impairment, as well as an increased likelihood of better self-rated health and mental well-being (44, 45).

#### 1.2.3 The mediating role of lifestyle

Education may help individuals adopt a healthy lifestyle, benefiting their overall well-being. According to the efficiency hypothesis of health production (3, 46, 47), well-educated individuals exhibit higher allocation efficiency in the health production function, enabling them to produce greater health. This efficiency may be realized through various aspects such as physical activity, diet, and smoking (3, 48). Based on previous findings (10, 25, 27), we further expand lifestyle to a multi-dimensional concept that includes six dimensions: overtime work, sleep, exercise, leisure, housework, and protein intake. The current study aims to examine whether different lifestyle dimensions mediate education attainment’s effects on health.

### 1.3 Objectives and hypotheses

This study has two aims: (1) to examine the relationship between educational attainment and self-rated health and depressive symptoms among Chinese adults and (2) to examine multi-dimensional lifestyles as mediators between educational attainment and self-rated health and depressive symptoms. We pose the following hypotheses: (H1) individuals with higher educational attainment are more likely to report higher levels of self-rated health and lower levels of depressive symptoms and (H2) multi-dimensional lifestyle factors, including exercise time, sleep time, overtime time, leisure time, housework time, and protein intake, mediate the association between educational attainment, self-rated health and depressive symptoms.

## 2 Methods

### 2.1 Study design and samples

The data were obtained from the first to the sixth wave of the China Family Panel Studies (CFPS) from 2012 to 2020. It was a nationwide longitudinal social survey that aimed to reflect the economic, demographic, educational, and health changes and their interactions among residents in China. The CFPS sample covered 31 provinces/municipalities/autonomous regions in mainland China. Because this study focused on the impact of educational attainment on the health status of Chinese adults, we excluded samples younger than 18 years of age. In addition, we also excluded samples of poor quality (e.g., missing information or anomalous values for critical variables, etc.). The analytic sample included 159,970 adults aged 18 years and older.

All participants provided written informed consent to participate in this study. The data used in this study were approved by the Biomedical Ethics Committee.

### 2.2 Measurements

#### 2.2.1 Outcome variables

##### 2.2.1.1 Self-rated health

Based on previous research (49), we used self-reported health (SRH) to measure people’s physical health. Self-reported health refers to people’s subjective assessment of their health, which was measured by asking respondents, “How would you rate your health?”; The responses to this question were “very poor,” “poor,” “fair,” “good,” and “very good,” which were assigned a score from 1 to 5, with larger scores indicating better health status. Self-rated health is a comprehensive assessment of an individual’s physical and psychological condition and has been widely used in health-related studies of Chinese adults (15).

##### 2.2.1.2 Depressive symptoms

Depressive symptoms were assessed with the Center for Epidemiological Studies Depression scale (CES-D) (50), which consists of 20 items ranging from one (rarely or never; less than 1 day) to four (most or all of the time; 5–7 days). Respondents reported the frequency of each item according to their psychological state and then summed the items to obtain a score ranging from 20 to 80. The higher the score, the more severe the respondent’s depressive symptoms. The scale was applied to the Chinese sample, proving high reliability and validity (51). This study’s reliability coefficient (Cronbach’s alpha value) was 0.93.

#### 2.2.2 Mediating variables

Lifestyle was measured by the amount of time a person spent working overtime, sleeping, exercising, leisure, doing housework each week/day, and protein intake, expanding previous research (10, 25, 27). Exercise time was the average weekly exercise frequency of the respondent. Housework time was measured by asking respondents, “Approximately how many hours do you spend doing housework at home every day?” Leisure time was measured by asking respondents how many hours they watched television, movies, and other video programs per week. Overtime was calculated by subtracting the regular scheduled working hours (usually 40 h per week in China) from the weekly total working hours. Sleep time was measured by asking respondents: “How many hours of sleep do you get each day?” Protein intake was measured by asking respondents: “In the past week, have you eaten meat (including pork, beef, lamb, chicken, duck, fish, shrimp, shellfish, etc.)?”

#### 2.2.3 Independent variable

Following Lynch’s method (5), educational attainment was measured by the years an individual has received education. Respondents were asked to answer their highest level of education based on the number of years of study they had completed.

#### 2.2.4 Confounding variables

Based on previous studies on education and physical and mental health (15, 52), we obtained the control variables for this study. They consisted of two main aspects: demographic characteristics and socioeconomic characteristics. The demographic characteristics variables mainly included gender (male, female), age (18–109), marital status (yes, no), members of the Communist Party of China (CPC) (yes, no), residence area (urban, rural), and geographic location (Western China, Central China, Eastern China). The socioeconomic variables comprised income level (low-income group, middle-income group, high-income group) and occupational types (professional technicians and managers, business service workers and office workers, production workers, farmers and the unemployed).

### 2.3 Statistical analysis

All data analysis was performed in Stata 15.1. Descriptive analysis was applied to present sample characteristics. In this case, mean and standard deviation were used to characterize continuous variables (e.g., age, educational attainment, depressive symptoms, and self-rated health), and frequency and percentage were used to characterize categorical variables (e.g., gender, marital status, CPC member, residence area, geographic location, income level, occupational types, and protein intake). The specific analytic process comprised three steps: first, we ran two multiple linear regression models to examine the relationship between educational attainment and self-rated health/depressive symptoms. Second, multiple linear regression models/binary logistic regression models were adopted to explore the association between educational attainment and lifestyle. Finally, based on the first step of the model, multidimensional lifestyle was further included in the model to test the mediating role of lifestyle in the associations between educational attainment and self-rated health, and educational attainment and depressive symptoms. The same covariates (age, gender, marital status, CPC member, residence area, geographic location, income level, occupational types) were incorporated into the model at each step.

## 3 Results

### 3.1 Descriptive analysis

Among 159,970 participants, the proportion of men and women was relatively evenly distributed, with both men and women accounting for approximately 50%. Most participants were married, and only 8.86% were members of the CPC (*n* = 13,964). Roughly 47.56% (*n* = 72,686) lived in urban areas. The average number of years of education for all participants was 7.209 years (range 0–22, SD = 5.079). For lifestyle, the average weekly overtime hours of the respondents was 24.119 (SD = 20.677), the average sleep time was 7.752 (SD = 1.521), the average leisure time was 10.971 (SD = 10.399), and the average housework time was 2.196 (SD = 2.009). More than half (*n* = 85,373, 55.76%) of the respondents exercised less than once a month, compared to about 26.95% (*n* = 41,270) who exercised seven times a week. The respondents’ age were 47.628 (range 18–109, SD = 16.633). Additionally, 85.16% (*n* = 75,915) of respondents consumed protein in the past week. More than 40% of the respondents were from Eastern China, with equal proportions (29%) from Central China and Western China. Regarding socioeconomic status variables, the distribution of respondents’ income was relatively even, with approximately 32% in the low-income and middle-income, respectively. In terms of occupation, the majority (60.63%) of respondents were farmers or unemployed, while professionals and managers accounted for only 8.56%. Among all respondents, the mean scores for self-rated health and depressive symptoms were 2.921 (range 1–5, SD = 1.24) and 33.088 (range 20–77, SD = 8.105), respectively, indicating that the respondents’ physical health and mental health conditions were all at a moderate level (Table 1).

**Table 1:** Descriptive analysis of sample characteristics.

	Mean	SD	Missing cases
**Self-rated health**	2.921	1.24	831 (0.52%)
**Depressive symptoms**	33.088	8.105	50342 (31.47%)
**Educational attainment**	7.209	5.079	744 (0.47%)
**Sleep time**	7.752	1.521	48563 (30.36%)
**Overtime**	24.119	20.677	100598 (62.89%)
**Leisure time**	10.971	10.399	48281 (30.18%)
**Housework time**	2.196	2.009	97986 (61.25%)
**Age**	47.628	16.633	11 (0.01%)
	**N**	**%**	
**CPC member**			2374 (1.48%)
Yes	13964	8.86	
No	143632	91.14	
**Marital status**			6430 (4.02%)
Yes	125603	81.80	
No	27937	18.20	
**Gender**			329 (0.21%)
Yes	79525	49.81	
No	80116	50.19	
**Residence area**			7155 (4.47%)
Urban	72686	47.56	
Rural	80129	52.44	
**Year**			0
2012	33306	20.82	
2014	34883	21.81	
2016	34527	21.58	
2018	32633	20.40	
2020	24621	15.39	
**Income level**			7812 (4.88%)
Low-income group	47932	31.50	
Middle-income group	48441	31.84	
High-income group	55785	36.66	
**Occupational type**			0
Professional technicians and managers	13689	8.56	
Business service workers and office workers	24090	15.06	
Production workers	25201	15.75	
Farmers and the unemployed	96990	60.63	
**Geographic location**			555 (0.35%)
Western China	46908	29.43	
Central China	46151	28.95	
Eastern China	66356	41.62	
**Protein intake**			70828 (44.28%)
Yes	75915	85.16	
No	13227	14.84	
**Exercise time**			6850 (4.28%)
Less than 0.25 times per week	85373	55.76	
More than 0.25 times but less than 1 time per week	3505	2.29	
1-2 times per week	9022	5.89	
3-4 times per week	9961	6.51	
5 times per week -6 times	2757	1.80	
7 times a week	41270	26.95	
More than 7 times a week	1232	0.80	

The values in parentheses represent the percentage of missing values among the 159,970 observations. Besides, the percentage of a specific category within a categorical variable refers to the ratio of that category among all valid responses for the variable, meaning that the calculation of percentages does not include missing values for the variable.

### 3.2 The association between educational attainment and health outcomes

Table 2 displays the results of multiple linear regression analysis of the relationship between educational attainment, self-rated health, and depressive symptoms. Model 1 indicated that educational attainment was significantly positively linked to higher levels of self-rated health after controlling for the relevant covariates (*B* = 0.006, *p* < 0.000). As for depressive symptoms, Model 2 revealed a significantly inverse association between educational attainment and depressive symptoms (*B* = −0.168, *p* < 0.000).

**Table 2:** Multiple linear regression analysis of the relationship between educational attainment, self-rated health, and depressive symptoms

	Model 1-self-rated health	Model 2- depressive symptoms
**Educational attainment**	0.006***	–0.168***
	(0.001)	(0.007)
**CPC member** (ref: no)	0.068***	–0.781***
	(0.011)	(0.088)
**Marital status** (ref: not married)	–0.018**	–2.188***
	(0.008)	(0.066)
**Gender** (ref: female)	0.203***	–1.344***
	(0.007)	(0.052)
**Age**	–0.023***	0.006***
	(0.000)	(0.002)
**Residence area** (ref: rural)	–0.053***	–0.650***
	(0.007)	(0.053)
Income level (ref: Low-income group)		
Middle-income group	0.117***	–1.248***
	(0.008)	(0.065)
High-income group	0.165***	–1.886***
	(0.009)	(0.072)
Occupational type (ref: professional technicians and managers)		
Business service workers and office workers	0.020	0.092
	(0.013)	(0.100)
Production workers	0.041***	0.215**
	(0.013)	(0.104)
Farmers and the unemployed	–0.019	–0.192**
	(0.013)	(0.098)
Geographic location (ref: western China)		
Central China	0.084***	–1.460***
	(0.008)	(0.065)
Eastern China	0.070***	–1.788***
	(0.008)	(0.061)
Year		
2014	0.199***	
	(0.009)	
2016	0.129***	–0.273***
	(0.009)	(0.067)
2018	0.155***	0.494***
	(0.010)	(0.069)
2020	0.233***	0.686***
	(0.011)	(0.076)
Constant	3.638***	39.068***
	(0.021)	(0.162)
Observations	140,389	103,581
R-squared	0.135	0.075

Standard errors in parentheses. ***p < 0.01, **p < 0.05, *p < 0.1.

### 3.3 Mediating effect of lifestyle on the association between educational attainment and health outcomes

Tables 3, 4 present the results of the causal steps approach (53) of the mediating effect of the lifestyle dimensions on the link between educational attainment and self-rated health, as well as educational attainment and depressive symptoms. Models 1–6 in Table 3 indicated that educational attainment showed a significant negative correlation with sleeping time (*B* = –0.007, *p* < 0.000), housework time (*B* = –0.011, *p* < 0.000), and overtime (*B* = –0.616, *p* < 0.000), as opposed to a significant positive correlation with exercise time (*B* = 0.061, *p* < 0.000), leisure time (*B* = 0.150, *p* < 0.000), and protein intake (*B* = 0.036, *p* < 0.000).

**Table 3:** Regression analysis of the relationship between educational attainment and lifestyle

	Model 1	Model 2	Model 3	Model 4	Model 5	Model 6
	Exercise time	Sleeping time	Overtime	Leisure time	Housework time	Protein intake
**Educational attainment**	0.061***	–0.007***	–0.616***	0.150***	–0.011***	0.036***
	(0.001)	(0.001)	(0.024)	(0.009)	(0.002)	(0.003)
**CPC member** (ref: no)	0.304***	–0.005	–2.874***	–0.162	–0.055*	0.172***
	(0.020)	(0.017)	(0.334)	(0.115)	(0.029)	(0.046)
**Marital status** (ref: not married)	–0.281***	–0.069***	1.907***	–0.676***	0.492***	0.678***
	(0.015)	(0.012)	(0.245)	(0.086)	(0.021)	(0.026)
**Gender** (ref: female)	–0.006	0.112***	3.394***	0.844***	–1.065***	–0.034
	(0.012)	(0.010)	(0.185)	(0.067)	(0.017)	(0.023)
**Age**	0.034***	–0.018***	–0.084***	0.086***	0.005***	0.007***
	(0.000)	(0.000)	(0.008)	(0.003)	(0.001)	(0.001)
**Residence area** (ref: rural)	0.352***	–0.149***	–1.788***	0.930***	–0.099***	0.487***
	(0.012)	(0.010)	(0.187)	(0.069)	(0.017)	(0.025)
Income level (ref: Low-income group)						
Middle-income group	0.116***	–0.115***	4.050***	1.074***	0.006	0.262***
	(0.015)	(0.012)	(0.247)	(0.085)	(0.020)	(0.028)
High-income group	0.268***	–0.134***	2.778***	1.278***	–0.162***	0.287***
	(0.017)	(0.014)	(0.263)	(0.093)	(0.022)	(0.032)
Occupational type (ref: professional technicians and managers)						
Business service workers and office workers	–0.018	–0.013	1.384***	1.318***	0.086**	–0.222***
	(0.024)	(0.018)	(0.324)	(0.123)	(0.036)	(0.058)
Production workers	–0.266***	0.019	0.322	0.691***	0.055	–0.663***
	(0.024)	(0.019)	(0.328)	(0.128)	(0.036)	(0.057)
Farmers and the unemployed	–0.020	0.035*	–0.601*	2.639***	0.681***	–0.160***
	(0.023)	(0.018)	(0.345)	(0.123)	(0.034)	(0.056)
Geographic location (ref: western China)						
Central China	–0.109***	–0.271***	0.278	0.769***	–0.339***	0.269***
	(0.015)	(0.012)	(0.228)	(0.084)	(0.021)	(0.028)
Eastern China	–0.171***	–0.365***	–0.530**	1.168***	–0.303***	0.497***
	(0.014)	(0.012)	(0.213)	(0.079)	(0.020)	(0.027)
Year						
2014	–0.156***		–12.153***			–0.002
	(0.017)		(0.250)			(0.026)
2016	0.184***	–0.056***	–10.160***	0.161*	–0.087***	
	(0.017)	(0.012)	(0.306)	(0.084)	(0.025)	
2018	0.233***	–0.112***	–10.513***	–1.117***	0.101***	
	(0.018)	(0.013)	(0.252)	(0.087)	(0.027)	
2020	–0.615***	–0.330***	–9.993***	–2.563***	0.264***	–0.338***
	(0.020)	(0.014)	(0.271)	(0.096)	(0.019)	(0.029)
Constant	0.713***	9.146***	35.140***	2.844***	1.962***	0.548***
	(0.038)	(0.030)	(0.601)	(0.206)	(0.051)	(0.077)
Observations	137,952	104,808	55,206	105,018	56,913	80,591
R-squared	0.089	0.056	0.086	0.042	0.149	

Standard errors in parentheses: ***p < 0.01, **p < 0.05, *p < 0.1; Models 1–5 used multiple linear regression analysis, while Model 6 used binary logistic regression analysis.

**Table 4:** Multiple linear regression analysis of the relationship between educational attainment, lifestyle, and health outcomes.

	Self-rated health						Depressive symptoms					
	Model 1	Model 2	Model 3	Model 4	Model 5	Model 6	Model 7	Model 8	Model 9	Model 10	Model 11	Model 12
**Educational attainment**	0.005***	0.004***	0.004***	0.004***	0.008***	0.008***	–0.154***	–0.159***	–0.149***	–0.149***	–0.159***	–0.178***
	(0.001)	(0.001)	(0.001)	(0.001)	(0.001)	(0.001)	(0.007)	(0.008)	(0.010)	(0.008)	(0.015)	(0.010)
**CPC member**(ref: no)	0.063***	0.061***	0.044**	0.060***	0.096***	0.079***	–0.713***	–0.713***	–0.490***	–0.713***	–0.866***	–0.889***
	(0.011)	(0.013)	(0.019)	(0.013)	(0.018)	(0.015)	(0.088)	(0.104)	(0.142)	(0.104)	(0.215)	(0.128)
**Marital status**(ref: not married)	–0.007	0.000	0.009	–0.002	–0.049***	–0.030***	–2.217***	–2.338***	–2.144***	–2.367***	–2.788***	–1.958***
	(0.008)	(0.010)	(0.014)	(0.010)	(0.013)	(0.011)	(0.066)	(0.077)	(0.106)	(0.077)	(0.157)	(0.099)
**Gender**(ref: female)	0.203***	0.200***	0.244***	0.206***	0.188***	0.206***	–1.342***	–1.235***	–1.470***	–1.262***	–0.937***	–1.290***
	(0.007)	(0.008)	(0.010)	(0.008)	(0.011)	(0.009)	(0.052)	(0.061)	(0.080)	(0.061)	(0.129)	(0.076)
**Age**	–0.024***	–0.022***	–0.022***	–0.022***	–0.023***	–0.024***	0.013***	–0.012***	0.016***	0.004*	–0.020***	0.006*
	(0.000)	(0.000)	(0.000)	(0.000)	(0.000)	(0.000)	(0.002)	(0.002)	(0.003)	(0.002)	(0.005)	(0.003)
**Residence area** (ref: rural)	–0.058***	–0.052***	–0.048***	–0.056***	–0.035***	–0.057***	–0.568***	–0.617***	–0.489***	–0.494***	–1.177***	–0.806***
	(0.007)	(0.008)	(0.011)	(0.008)	(0.011)	(0.009)	(0.054)	(0.063)	(0.080)	(0.063)	(0.126)	(0.079)
Income level (ref: Low-income group)												
Middle-income group	0.114***	0.110***	0.121***	0.106***	0.103***	0.110***	–1.218***	–1.155***	–1.189***	–1.042***	–0.830***	–1.258***
	(0.008)	(0.010)	(0.014)	(0.010)	(0.013)	(0.011)	(0.064)	(0.078)	(0.108)	(0.078)	(0.155)	(0.094)
High-income group	0.160***	0.156***	0.154***	0.151***	0.182***	0.167***	–1.828***	–1.838***	–1.557***	–1.702***	–1.965***	–1.913***
	(0.009)	(0.010)	(0.015)	(0.011)	(0.014)	(0.012)	(0.072)	(0.084)	(0.115)	(0.085)	(0.160)	(0.107)
Occupational type (ref: professional technicians and managers)												
Business service workers and office workers	0.020	0.012	0.030	0.014	0.012	0.026	0.089	0.194*	0.034	0.255**	0.008	–0.037
	(0.013)	(0.014)	(0.018)	(0.014)	(0.022)	(0.018)	(0.100)	(0.108)	(0.137)	(0.108)	(0.265)	(0.162)
Production workers	0.042***	0.028*	0.041**	0.027*	0.029	0.055***	0.151	0.367***	0.210	0.376***	0.488*	0.101
	(0.013)	(0.014)	(0.018)	(0.014)	(0.023)	(0.018)	(0.104)	(0.112)	(0.140)	(0.113)	(0.269)	(0.167)
Farmers and the unemployed	–0.018	–0.036***	0.011	–0.033**	–0.030	0.012	–0.201**	0.071	0.113	0.136	–0.191	–0.435***
	(0.013)	(0.014)	(0.019)	(0.014)	(0.021)	(0.017)	(0.098)	(0.109)	(0.147)	(0.110)	(0.252)	(0.155)
Geographic location (ref: western China)												
Central China	0.087***	0.099***	0.093***	0.089***	0.093***	0.086***	–1.481***	–1.183***	–1.375***	–0.969***	–0.878***	–1.916***
	(0.008)	(0.010)	(0.013)	(0.009)	(0.013)	(0.011)	(0.065)	(0.076)	(0.098)	(0.076)	(0.154)	(0.095)
Eastern China	0.078***	0.092***	0.101***	0.079***	0.085***	0.072***	–1.822***	–1.713***	–1.812***	–1.447***	–1.318***	–2.058***
	(0.008)	(0.009)	(0.012)	(0.009)	(0.012)	(0.010)	(0.061)	(0.072)	(0.092)	(0.072)	(0.146)	(0.091)
Year												
2014	0.216***		0.201***			0.203***						
	(0.010)		(0.014)			(0.009)						
2016	0.140***	–0.072***	0.156***	–0.073***	–0.218***		–0.293***		0.788***			
	(0.009)	(0.010)	(0.017)	(0.010)	(0.016)		(0.067)		(0.123)			
2018	0.166***	–0.049***	0.144***	–0.052***	–0.190***		0.530***	0.750***	1.202***	0.721***		
	(0.010)	(0.010)	(0.014)	(0.010)	(0.017)		(0.069)	(0.066)	(0.098)	(0.066)		
2020	0.259***	0.040***	0.225***	0.027**	0.030**	0.239***	0.553***	0.791***	1.468***	0.843***	–0.325**	0.454***
	(0.011)	(0.011)	(0.016)	(0.011)	(0.012)	(0.011)	(0.076)	(0.073)	(0.106)	(0.074)	(0.132)	(0.081)
**Exercise time**	0.018***						–0.214***					
	(0.001)						(0.012)					
**Sleeping time**		0.036***						–0.595***				
		(0.002)						(0.019)				
**Overtime**			–0.001***						0.020***			
			(0.000)						(0.002)			
**Leisure time**				–0.001						–0.040***		
				(0.000)						(0.003)		
**Housework time**					0.021***						0.100***	
					(0.003)						(0.029)	
**Protein intake** (ref: no)						0.057***						–1.911***
						(0.012)						(0.139)
Constant	3.601***	3.487***	3.564***	3.821***	3.775***	3.597***	39.198***	43.923***	37.304***	38.608***	41.148***	41.344***
	(0.021)	(0.032)	(0.035)	(0.023)	(0.032)	(0.028)	(0.162)	(0.254)	(0.265)	(0.183)	(0.415)	(0.274)
Observations	137,632	104,797	55,028	105,007	56,906	80,548	103,574	75,900	41,389	75,960	20,721	46,705
R-squared	0.134	0.119	0.096	0.117	0.139	0.141	0.078	0.076	0.070	0.067	0.070	0.090

Standard errors in parentheses. ***p < 0.01, **p < 0.05, *p < 0.1.

Model 3–4 in Table 4 showed that leisure time was not significantly related to self-rated health, and overtime work was negatively associated with self-rated health (*B* = –0.001, *p* < 0.000). In contrast, sleeping time, exercise time, housework time, and protein intake all indicated a significant positive relationship with self-rated health (*B* = 0.036, *p* < 0.000; *B* = 0.018, *p* < 0.000; *B* = 0.021, *p* < 0.000; *B* = 0.057, *p* < 0.000). In addition, the indirect effect values for time spent exercising (0.061*0.018), overtime (–0.616*–0.001), and protein intake (0.036*0.057) were in the same direction as the direct effect values for their respective educational attainment (*B* = 0.005; *B* = 0.004; *B* = 0.008; see model 1, model 3, and model 6 in Table 4). That means that exercise time, overtime, and protein intake mediated the relationship between educational attainment and self-rated health. However, the indirect effect value for sleeping time (–0.007*0.036) was in the opposite direction to the direct effect value for educational attainment (*B* = 0.004, see model 2), as was the indirect effect value for housework time (–0.011*0.021) and the corresponding direct effect value for educational attainment (*B* = 0.008, see model 5 in Table 4).

The results of Models 7–12 in Table 4 demonstrated that exercise time, sleeping time, leisure time, and protein intake all exhibited an important adverse correlation with depressive symptoms (*B* = −0.214, *p* < 0.000; *B* = −0.595, *p* < 0.000; *B* = −0.040, *p* < 0.000; *B* = −1.911, *p* < 0.000), while overtime and housework time were both exhibited a significant positive correlation with depressive symptoms (*B* = 0.020, *p* < 0.000; *B* = 0.100, *p* < 0.000). Moreover, the indirect effect values for exercise time (0.061*–0.214), leisure time (0.150*–0.040), housework time (–0.011*0.1), overtime (–0.616*0.020), and protein intake (0.036*–1.911) were in the same direction as the respective direct effect values for educational attainment (*B* = –0.154; *B* = –0.149; *B* = –0.159; *B* = –0.149; *B* = –0.178; see models 7–12 in Table 4), while the direction of the indirect effect for sleeping time (–0.007*–0.595) and the direction of the direct effect of educational attainment were reversed (*B* = –0.159, see model 8 in Table 4). That is, exercise time, leisure time, housework time, overtime time, and protein intake all normally mediated the relationship between educational attainment and depressive symptoms, while sleep time acted as a suppressive effect (54) in the above relationship.

## 4 Discussion

This study examined the relationship between educational attainment and health outcomes, with multidimensional lifestyle factors as mediators. Our empirical results showed that individuals with higher educational attainment had better physical and mental health, supporting H1. In line with hypothesis 2, we found that various lifestyle dimensions (exercise time, sleep time, overtime, housework time, and protein intake) mediated the relationship between educational attainment and self-rated health, as well as educational attainment and depressive symptoms. Also, leisure time mediated the association between educational attainment and depressive symptoms but not the association between educational attainment and self-rated health, which partially supported H2. Specifically, educational attainment improved individuals’ self-rated health and reduced depressive symptoms by reducing overtime work hours, increasing exercise time, and enhancing protein intake. Meanwhile, elevated educational attainment reduced individuals’ sleep time, leading to lower self-rated health and higher levels of depressive symptoms. Additionally, the reduced housework time and more leisure time mediate higher educational attainment’s associations with lower depressive symptoms, but not with better self-rated health.

Consistent with previous research (5, 6, 13), higher educational attainment significantly promoted individuals’ self-rated health and reduced depressive symptoms, and these findings hold true across most income groups. The link between educational attainment and physical and mental health could be attributed to several aspects. According to the human capital theory of education (55), receiving an education is the process of stockpiling human capital and developing productive capacity. Thus, the longer an individual gets an education, the more human capital is gained, i.e., more knowledge, skills, and competencies are possessed, which can promote health. Hummer et al.’s study showed that those with 12 years of education had a 21% lower mortality rate than those with eight or fewer years but a 33% higher mortality rate than those with 17 or more years (6). From the perspective of the fundamental cause theory in sociology, SES is considered a fundamental determinant of health (56). Education is the primary and core dimension of SES (55) and may have become a reproducer of inequality. Higher education reflects an individual’s socioeconomic resource advantage within the social structure. The upper social classes can translate the socioeconomic resource advantage they possess into a health advantage, which creates a health gradient between the upper and lower social classes (those with lower SES) due to differences in the resources they have access to (57). Numerous studies demonstrated the education-health gradient, whereby higher levels of education are associated with better physical health (e.g., lower mortality, etc.) and better psychological states (e.g., less depressive emotion, etc.) (13, 58).

Moreover, increased exercise time, higher protein intake, and reduced overtime work were critical ways that educational attainment improved self-rated health and decreased depressive symptoms, which could be understood in the following ways. According to the human capital perspective of learned effectiveness (10), greater problem-solving abilities enable education to guide individuals to become active and effective agents in their own lives and cultivate beliefs in control over their lives. This motivation encourages people to integrate health-promoting behaviors into a coherent lifestyle (10), which becomes an effective means for highly educated individuals to achieve their health. Higher levels of education contribute to improving individuals’ health literacy, enhancing their ability to comprehend health information, and consequently encouraging individuals to adopt more proactive health behaviors (59). A previous study found that participants with higher education exhibited significantly greater adherence to dietary guidelines than those with primary education (23). Besides, well-educated individuals are more likely to allocate more leisure time to physical activity on weekends/holidays (25, 60). These activities are particularly beneficial in reducing diseases and alleviating emotional distress, such as depression and anxiety (61).

Furthermore, working hours may contribute to health inequalities due to class differences in non-standard working hours. A recent study showed that individuals with lower levels of education are more likely to work outside of standard working hours due to their lower autonomy in time control (28). It is worth noting that long working hours (e.g., more than 69 h per week) have been established as predictors of diabetes, musculoskeletal disorders, and severe depression (33, 62). The perspective of class distinction (63–65) also helps to understand this mechanism. Class status and its associated resources lead to health inequality through the habitus mechanism (66). Lifestyle is a habitual tendency formed through the interaction of capital and field, reflecting the behavioral patterns of specific social classes (65). Higher social classes have more time and resources to adopt the healthiest lifestyles (65). For instance, Bourdieu observed that class-oriented consumption habits shape the differentiated dietary practices and exercise preferences across social classes: the middle and upper classes preferred less fatty, light, low-calorie, and high-protein foods (e.g., beef and lamb, fresh fruits and vegetables) to shape their bodies, while the working class favored cheaper, calorie-dense, nutritious foods (e.g., potatoes, cabbage, and pork) to maintain a sense of strength in the male body (63). Moreover, activities that represented the refined cultural tastes of higher cultural capital classes, such as moderate weight training, intense aerobic exercise, and avoiding physically dominating competitive sports, were more commonly found among the upper classes (67). All these factors directly or indirectly contributed to improved health outcomes (10, 33). Hence, it is not difficult to understand the gradient between exercise time and overtime hours in health in cultural capital (education). Moreover, the longevity benefits perspective based on cost–benefit principles (13, 68) also provides a valuable explanation: education can offer individuals a better future, such as higher income and increased happiness. Therefore, highly educated individuals are more willing to invest in health (e.g., engaging in health behaviors) to protect these future gains. In contrast, individuals with lower levels of education have a lower future value of living to old age (i.e., less benefit from longevity) and may be more likely to focus on the present, engaging in health-risk behaviors. For low-educated individuals, the rewards of healthy behaviors are limited (because of shorter life expectancy), and instead, indulging in unhealthy but enjoyable behaviors may be more meaningful (64).

Also, decreased sleeping time resulting from higher educational attainment suppressed the positive effects of educational attainment in improving self-rated health and reducing depressive symptoms, contrary to the role of sleep in previous studies (69). Concerning the first pathway - the negative correlation between higher educational attainment and sleep hours - could be explained by the substitution effect at work (60). From an economic perspective of opportunity cost, in situations where the total available time is limited, individuals with higher levels of education are more productive. Their value in the labor market (wage rate) is higher, which may lead to higher opportunity costs for individuals with higher educational levels in allocating sleep time (64). An earlier study found that both more educated men and women allocated less time to certain health-promoting activities such as sleep and that this was worse at work than on weekends (60). The second pathway pertains to the relationship between sleeping time, self-rated health, and depressive symptoms. Several systematic reviews suggest that sleep deprivation (e.g., less than 7 h of sleep per day) may affect health-related quality of life, such as an increased risk of cardiovascular and Alzheimer’s disease, cognitive decline, and may lead to depression and phobias (36, 70).

More interestingly, increases in leisure time and reductions in housework time resulting from higher educational attainment hold different effects on self-rated health and depressive symptoms. Firstly, for the first path - the positive effects of higher educational attainment on increased personal leisure time and reduced housework time - the previously mentioned theories of learned effectiveness (10) and class distinction (63–65) still contribute to the understanding of this issue and will not be repeated and expanded upon here. A cross-sectional study focusing on respondents aged 25–64 in the United States concluded that those with higher levels of education spent less time on personal/family activities during the workday due to the work substitution effect (60). Another study illustrated that certain leisure activities (e.g., going to the movies or watching a play) were perceived as expressions of high culture tastes and were more likely to be part of the daily leisure activities of those with higher cultural capital (71). Secondly, one of the dimensions of the second pathway—the association between housework time/leisure time and self-rated health/depressive symptoms—will be discussed separately.

Concerning the role of housework time in self-rated health, a large number of studies have confirmed the critical role of housework participation in improving individuals’ self-rated health and decreasing the risk of disease (34, 39, 40), and our study further supports these viewpoints. Household labor participation may have beneficial effects similar to physical activity, thus compensating for the drawbacks of insufficient exercise on physical health, and by participating in housework, individuals can succeed in their family roles and enhance their sense of self-worth (42), which may be beneficial to individual health. The cognitive benefits of housework may also contribute to an individual’s quality of life (e.g., brain health) (72). Thus, while higher educational attainment assists individuals in getting rid of burdensome housework, it concurrently suppresses their physical well-being. However, regarding the role of housework in mental health, as in previous studies (41), less housework is associated with fewer depressive symptoms. According to the Work–Family Conflict Model and Role Conflict Theory (73, 74), when individuals simultaneously experience multiple role pressures in the domains of work and family and lack the (temporal or psychological) resources to fulfill these roles, this situation may result in individuals experiencing role overload and role conflict, potentially leading to various physical and psychological symptoms. Thus, the reduced housework resulting from higher educational attainment may contribute to alleviating the “burden” in family life, reducing the stress of daily life, and avoiding role overload, thus aiding in reducing depressive symptoms.

As for the other dimension of the second pathway—the link between leisure time and self-rated health and depressive symptoms—the following study may help to understand it. For depressive symptoms, consistent with previous research (75), more leisure time may help to reduce individuals’ depressive symptoms. Individuals may resort to watching television to escape or alleviate real-life stress or problems (75). Short-term avoidance behavior may be adaptive as an avoidance coping strategy in a given situation, as it helps to divert attention from excessive personal stress, thereby reducing psychological distress (76). Regarding self-rated health, the association between leisure time and self-rated health was insignificant, implying that leisure time did not mediate the relationship between educational attainment and self-rated health. This may be because, recreational leisure activities, such as watching TV, imply prolonged sedentary behavior and reduced physical activity (77), which may lead to health risks such as obesity, type 2 diabetes, and cardiovascular disease (77). Besides, sedentary leisure activities like watching TV may involve an increased high-density energy intake (38), and these unhealthy diets may increase the potential risk of future disease.

### 4.1 Limitations and implications

There are limitations in our study. On the one hand, the measurement of educational attainment was limited to years of formal schooling and did not include other types of education such as vocational schools. On the other hand, our assessment of lifestyle factors was not sufficiently comprehensive due to inconsistent measurement across waves, particularly as it did not consider health risk behaviors such as smoking and drinking. This limited a deeper exploration of the role of lifestyle factors in the health returns to education.

Despite these limitations, our study provides valuable insights. First, we find that educational attainment can promote individuals’ physical and mental well-being. Therefore, the government should guarantee its citizens fair access to education, continuously increase its investment in public education, and improve the quality of education to enhance their human capital. Second, lifestyle is a significant mediating mechanism. Therefore, schools should incorporate health education into their regular curriculum, disseminating and broadening students’ health knowledge and assisting them in developing healthy lifestyle habits.

## 5 Conclusion

This study demonstrates that educational attainment is associated with elevated self-rated health and decreased depressive symptoms among Chinese adults. Educational attainment promoted physical and mental health by increasing exercise time, reducing overtime, and improving protein intake. Our findings also indicate that the adverse consequence associated with educational attainments, such as reduced sleeping time, might suppress the positive effects of higher educational attainment on better health outcomes. Moreover, our research reveals that other dimensions of lifestyle—leisure time and housework time—might mediate differently between educational attainment and self-rated health, as well as educational attainment and depressive symptoms, respectively. These findings provide crucial insights for the government to reduce health inequalities in the future.

## Data Availability

The raw data supporting the conclusions of this article will be made available by the authors, without undue reservation.
